# Cr_2_S_3_-Cr_2_O_3_/Poly-2-aminobenzene-1-thiol as a Highly Photocatalytic Material for Green Hydrogen Generation from Sewage Water

**DOI:** 10.3390/mi14081567

**Published:** 2023-08-07

**Authors:** Mohamed Rabia, Asmaa M. Elsayed, Maha Abdallah Alnuwaiser

**Affiliations:** 1 Nanomaterials Science Research Laboratory, Chemistry Department, Faculty of Science, Beni-Suef University, Beni-Suef 62514, Egypt; mohamedchem@science.bsu.edu.eg; 2 TH-PPM Group, Physics Department, Faculty of Science, Beni-Suef University, Beni-Suef 62514, Egypt; 3 Department of Chemistry, College of Science, Princess Nourah bint Abdulrahman University, P.O. Box 84428, Riyadh 11671, Saudi Arabia

**Keywords:** Cr_2_S_3_, Cr_2_O_3_, poly-2-aminobenzene-1-thiol, sewage water, green hydrogen, renewable energy

## Abstract

This study highlights the utilization of the Cr_2_S_3_-Cr_2_O_3_/P2ABT nanocomposite photoelectrode for efficient and highly sensitive photon absorption, enabling the generation of green hydrogen through the production of hot electrons upon illumination. The nanocomposite is synthesized via a one-pot reaction using K_2_Cr_2_O_7_ and 2-aminobenzene-1-thiol monomer, and the presence of Cr_2_S_3_-Cr_2_O_3_ is confirmed by XRD and XPS analysis within the composite. The optical properties of the Cr_2_S_3_-Cr_2_O_3_/poly-2-aminobenzene-1-thiol composite exhibit wide spectral coverage from UV to IR, with a bandgap of 1.6 eV. The diverse morphological behavior observed in the composite correlates with its optical properties, with the cleft spherical particles of the pure polymer transforming into rod-like structures embedded within the polymer matrix. The generated hydrogen gas demonstrates an impressive efficiency of 40.5 mole/10.cm^2^.h through electrochemical testing. The current density (J_ph_) values are evaluated under different light frequencies using optical filters ranging from 730 to 340 nm, resulting in Jph values of 0.012 and 0.014 mA.cm^−2^, respectively. These findings present a promising avenue as green hydrogen for industrial applications, leveraging the potential of the Cr_2_S_3_-Cr_2_O_3_/P2ABT nanocomposite photoelectrode.

## 1. Introduction

One of the vital matters for clean energy technology in the future is the treatment of wastewater by producing photoactive hydrogen using solar energy. This technology will be readily available as an environmentally friendly and low-cost technology in addition to improving other energy sources. An attractive method for water dispersion photocatalysis is a newly emerging approach to improve energy conversion technology [1,2,3]. From different types of energy production, hydrogen is a clean and environmentally friendly energy source, with high energy combustion and zero carbon emission [4]. Solar energy has good applications in the production of electricity and hydrogen gas. The dispersion of water to hydrogen relies on the use of photocatalytic semiconductors that have the ability to absorb light to produce electrons on the water molecules that react to give H_2_ and O_2_ gases. H_2_ gas is a fuel with large combustion energy and can be used as a fuel for rockets and spacecraft [5,6,7]. Recently, the idea of a solar-activated photocatalytic dewatering technique as a method to produce hydrogen gas from wastewater. Inorganic photocatalysts are used as photo absorbers to produce electron-hole pairs, while the photocatalysts reduce and oxidize water to give hydrogen and oxygen. Metal photo supports are in great demand but are high in cost, corrosive, and potentially hazardous. Increasing the surface area (through nanoparticles) is one of the ways to improve the activity of photosynthesis [8,9]. Materials that have the ability to reach the state of plasma or that have a tolerance to high temperatures are considered a good choice to increase photo-support activity, such as noble or active metal. These metals absorb light well, allowing electron doping via the composite semiconductor, which is used by neighboring materials to produce hydrogen. Conductive polymers offer the properties of low cost, ease of synthesis, outstanding electrochemical activity, good electrical conductivity, and fast carrier mobility. Conjugated polymers, as a new type of photo protectant, are very active in the effect of UV and visible light. These organic polymers have made progress in photo support and solar energy conversion. Polypyrrole (PPY), polyaniline and its derivatives, and polythiophene and its derivatives are the main categories of conjugated polymers, which have been extensively explored and applied as ultracapacitor electrodes and tumor cell ablation.

Previous studies have explored the use of other materials, such as PANI/TiO_2_ and PANI/MoS_2_, as catalytic electrodes for hydrogen. However, the efficiency achieved in these studies has been very limited, with almost negligible results. The resulting J_ph_ values were in the microampere range, indicating a significant challenge in achieving substantial hydrogen generation [10]. To address this issue, other studies have attempted to enhance the performance by employing materials such as Ni/PANI or poly(3-aminobenzoic acid). However, the problem of limited efficiency still persists, as indicated by previous investigations.

Among the organic photo supports, polymer/metal oxide, one of the most promising crosslinked polymers in the photo-support field, can be synthesized by chemical methods, by radioactive reaction of water, and by using templates such as hexagonal meso-phases. All studies reported still suffer from a low amount of hydrogen produced and low efficiency of maybe 2%. Furthermore, previous studies used methods to obtain hydrogen gases that induced electrode corrosion. Studies also used fresh water as a source of hydrogen gas without taking into account the lack of fresh water for drinking in many countries of the world. However, some previous studies used complex devices such as atomic layer deposition or plasma deposition to precipitate metal oxides or sulfides [2,11].

In this study, the potential of Cr_2_S_3_-Cr_2_O_3_/poly-2-aminobenzene-1-thiol (P2ABT) as a highly photocatalytic material for green H_2_ generation from sewage water is investigated. We conducted a comprehensive analysis to confirm the elemental composition, crystalline structure, functional groups, topography, and morphological behavior of the nanocomposite. The green hydrogen production was applied through a three-electrode cell setup. The efficiency of hydrogen production was evaluated based on the J_ph_ generated, and the amount of hydrogen produced was calculated using the Faraday electrolysis law. We conducted the evaluation over a wide range of optical spectra, spanning from 340 to 730 nm.

## 2. Materials and Methods

### 2.1. Characterizations and Used Materials

The chemicals K_2_Cr_2_O_7_ and HCl were acquired from Winlab (Watford, UK), whereas 2-aminobenzene-1-thiol and dimethylformamide were obtained from Merck (Darmstadt, Germany) and Sigma-Aldrich (St. Louis, MO, USA), respectively. El-Naser Chemical Company is the source of K_2_S_2_O_8_ in Egypt. The third stage wastewater, which was treated and came with a complete chemical composition, was provided by the drinking water company in Beni Suef, Egypt.

The materials underwent morphological analyses using TEM and SEM and theoretical surface modeling that was conducted using the tools JEOL, ZEISS, and Gwydion, respectively. The optical analysis was investigated by a spectrophotometer (Birkin Elmer, Wellesley, MA, USA). Chemical composition, structure, crystallinity, and elemental analyses were determined using FTIR from Bruker (Carteret, NJ, USA), XRD from PANalytical Pro (Malvern, UK), and XPS from Jasco(Waltham, MA, USA).

### 2.2. P2ABT and Cr(III)oxide/P2ABT Photoelectrode Preparation

The oxidation polymerization process was utilized for the synthesis of either pure P2ABT or Cr(III) oxide/P2ABT on glass slides. In this process, the 2-aminobenzene-1-thiol monomer was oxidized using K_2_S_2_O_8_ and K_2_Cr_2_O_7_ to obtain pure P2ABT and Cr(III) oxide/P2ABT, respectively. The monomer and the oxidant were mixed in an aqueous solution with concentrations of 0.12 M and 0.15 M, respectively, and stirred. The use of K_2_Cr_2_O_7_ as an oxidant resulted in the incorporation of Cr^3+^ oxide as a dopant material into the polymer. HCl was used as an acid medium, solvent, and dopant for the polymer. After complete polymerization, the formed P2ABT or Cr(III) oxide/P2ABT films were obtained, treated, and dried.

### 2.3. The Hydrogen Generation through the Electrochemical Wastewater Splitting Reaction

The wastewater with the chemical composition specified in Table 1 is utilized as a source of hydrogen, with heavy metals and other suspended elements serving as sacrificial agents (pH > 7). The photoelectrochemical reaction is conducted using CHI608E as the electrolyte. The J_ph_-V and J_ph_-time relationships are evaluated under photon illumination (halide lamp) to identify the hydrogen generation reaction. The J_ph_ value generated during the wastewater splitting process indicates the efficiency, moles, and rate of H_2_ gas production.

## 3. Results and Discussion

### 3.1. Analyses

**Figure 1:**
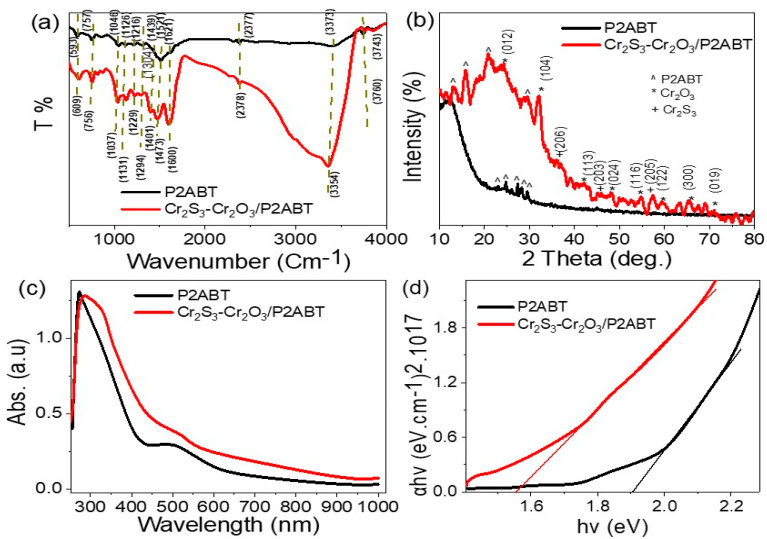
Chemical characterization of P2ABT and Cr_2_S_3_-Cr_2_O_3_/P2ABT nanocomposite: (**a**) FTIR and (**b**) XRD. The optical behavior of these materials: (**c**) absorbance and (**d**) bandgap.

According to the FTIR analysis shown in Figure 1a, the function groups present in both P2ABT and Cr_2_S_3_-Cr_2_O_3_/P2ABT nanocomposite are confirmed, but with small shifts due to the presence of inorganic Cr_2_S_3_-Cr_2_O_3_ in the polymer matrix [13]. Table 1 summarizes the position of the function groups and their shift behavior in the nanocomposite compared to the pure P2ABT.

The XRD pattern indicates the crystalline nature of both P2ABT and Cr_2_S_3_-Cr_2_O_3_/P2ABT nanocomposite, as shown in Figure 1b. The sharp peaks observed in the 2theta range of 22.7°, 24.9°, 27.4°, 28.4°, and 29.7° for pure P2ABT are an indication of its crystallinity, which is a favorable property for optical applications.

The behavior of the P2ABT crystal within the composite differs from that of the pure polymer, as indicated by the emergence of new peaks ranging from 13.2° to 29.3°, represented by four distinct peaks. These shifts in peaks are attributed to the interaction and incorporation of inorganic materials within the polymer matrix.

In the composite, the presence of Cr_2_O_3_ is identified by characteristic peaks observed at eight specific positions: 24.4°, 32.3°, 42.1°, 48.3°, 54.7°, 59.8°, 65.5°, and 71.4°. Each of these peaks corresponds to the growth directions of (012), (104), (113), (024), (116), (122), (300), and (019), respectively, as defined by the JCPDS-038-1479 reference [14].

Additionally, Cr2S3 is observed in the composite, with peaks located at 36.6°, 45.4°, and 57.4°, representing the growth directions (206), (205), and (203) according to the JCPDS-011-0007 reference [15].

The Cr_2_S_3_-Cr_2_O_3_/P2ABT nanocomposite exhibits a wide optical absorbance ranging from 250 to 800 nm, indicating high absorbance across a broad spectrum (Figure 1c). This behavior is related to the combination of the organic (P2ABT) and inorganic (Cr_2_S_3_-Cr_2_O_3_) components, which act together to capture photons and promote the crystal structure of the nanocomposite. The polymer P2ABT has a high photon response in the UV and Vis regions under the electron transitions within the polymer chain, specifically the bi-bi* transition [16,17].

The bandgap of the nanocomposite is significantly reduced after the formation of the composite, from 1.95 to 1.6 eV. This reduced bandgap value is considered ideal for the composite, enabling it to function in any optical region. Moreover, the material is also affected by temperature, causing electron vibration and the generation of additional electrons.

The evaluation of bandgap values is illustrated using optical parameters such as absorbance coefficient (α) and light frequency, in which the absorbance (A) is considered a main parameter for this evaluation through the mathematical equation (Tauc’s equation (Equation (1))) [18].

(1) $$\alpha h\nu \;=\;A{\left(h\nu -E_{g}\right)}^{1/2}$$

**Figure 2:**
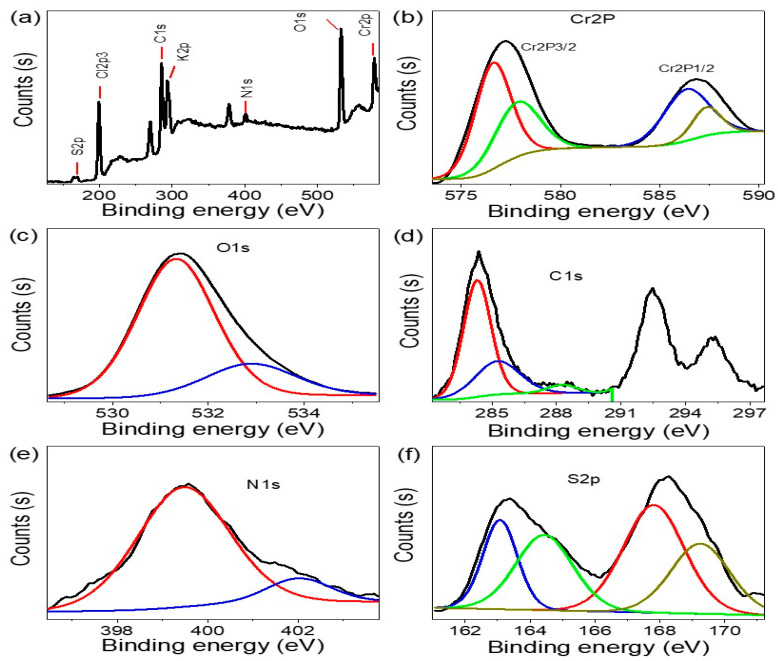
The XPS analyses: (**a**) survey, (**b**) Cr2p, (**c**) O1s, (**d**) C1s, (**e**) N1s, and (**f**) S2p for the Cr_2_S_3_-Cr_2_O_3_/P2ABT nanocomposite.

The XPS analysis (Figure 2) of the Cr_2_S_3_-Cr_2_O_3_/P2ABT nanocomposite shows the presence of all elements related to the P2ABT polymer, including C1s, N1s, and S2p, which are located at 285.1, 400.5, and 166 eV, respectively. The presence of multiple peaks for the C element in the XPS spectrum is due to the formation of various bonds, such as C-C, C=C, C-O, and C-S, during the synthesis of the composite. These bonds are formed through the interaction between the organic and inorganic components of the composite. Additionally, the presence of Cr_2_S_3_-Cr_2_O_3_ in the nanocomposite is confirmed through the appearance of Cr2p spectra, specifically, Cr2p3/2 at 576.4 eV, which is related to Cr_2_O_3_. Other peaks located at 578 eV are related to traces of CrO_3_ [19,20], in which the Cr and O element percent is 4.3 and 21% in the nanocomposite. The peaks at 586 eV support the formation of Cr(III) compounds [21]. Moreover, the peaks related to S are located at 162.7 eV (S2p3/2) and 167.4 eV (S2p1/2), indicating the formation of Cr_2_S_3_ within the polymer matrix, the composite has an S element percent of 0.5%. These findings suggest a successful formation of the Cr_2_S_3_-Cr_2_O_3_/P2ABT nanocomposite.

**Figure 3:**
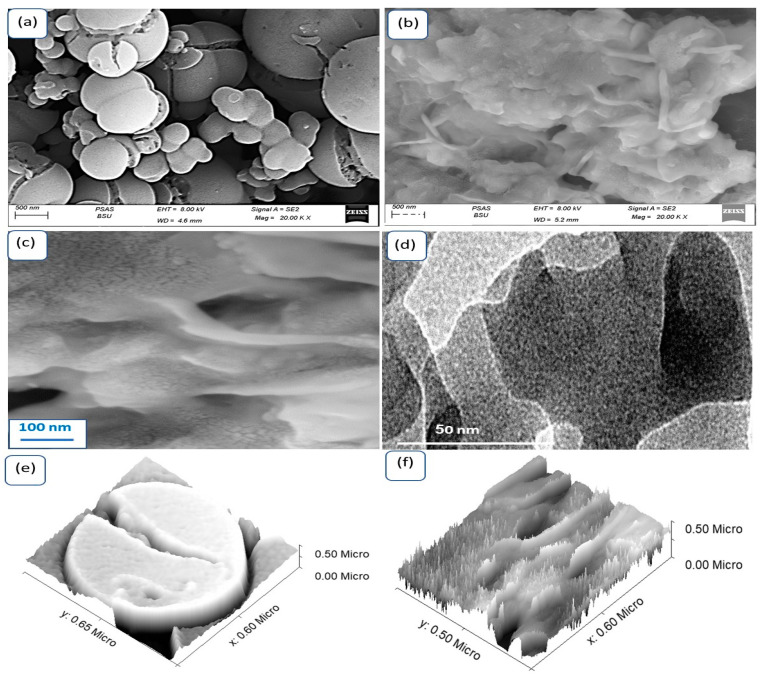
The SEM analyses of (**a**) P2ABT and (**b**,**c**) Cr_2_S_3_-Cr_2_O_3_/P2ABT nanocomposites under various magnifications. (**d**) TEM of Cr_2_S_3_-Cr_2_O_3_/P2ABT nanocomposite. The roughness and cross-section of (**e**) P2ABT and (**f**) Cr_2_S_3_-Cr_2_O_3_/P2ABT nanocomposites.

The morphological characteristics of the Cr_2_S_3_-Cr_2_O_3_/P2ABT nanocomposite exhibit greater diversity compared to the individual particles of P2ABT, as shown in Figure 3. In Figure 3a, the P2ABT particles are observed to have a cleft or ball-shaped morphology. In contrast, the nanocomposite (Figure 3b) displays rod-like structures embedded within spherical particles. This unique morphology allows the composite to gather the smaller particles together, forming a single shape that can be utilized to create thin films, making it advantageous for photocatalytic applications.

Figure 3c depicts the close contact between the polymer and these rod-like structures, enabling the particles to function collectively for efficient photon capture in optoelectronic applications. The TEM image in Figure 3d clearly demonstrates this behavior, with particles agglomerating and effectively coating the rod-shaped structures of the Cr_2_S_3_-Cr_2_O_3_/P2ABT nanocomposite.

Theoretical modeling of the P2ABT and Cr_2_S_3_-Cr_2_O_3_/P2ABT nanocomposite is presented in Figure 3e,f, respectively. The pure polymer exhibits a cleft spherical morphology, while the composite showcases the rods embedded within the polymer particles. The rods have a thickness of 20 nm and a length of 450 nm, as determined by the theoretical modeling.

### 3.2. The Electrochemical Sewage Water Splitting for Hydrogen Gas Generation

Electrochemical sewage water splitting is a promising method for generating hydrogen gas, and the high-efficiency photoelectrode Cr_2_S_3_-Cr_2_O_3_/P2ABT nanocomposite has been studied for this purpose using a three-electrode cell. The generation of H_2_ gas is related to the efficiency of the photoelectrode, which is sensitive to photons due to the multi-component construction of the photoelectrode. The nanocomposite is capable of absorbing photons over a wide range of the optical spectrum, ranging from UV to IR, and can induce electron transition or vibration to promote the efficient splitting of water molecules. Electrolysis of sewage water is a promising field of study as it utilizes an abundant and easily accessible source of electrolytes. The chemical composition and heavy metal content of sewage water are shown in Table 2. Heavy metals in the solution are believed to play a significant role as sacrificial agents in the generation of hydrogen gas due to their high mobility and chemical reactivity. This role may be enhanced in the presence of light, where photon excitation of the photoelectrode may further facilitate the reactions involving these heavy metals.

The energy of photons has a direct impact on the generation of electrons, which are reflected in the form of J_ph_ values observed in the generated hydrogen gas. The hot electrons are sited on the photoelectrode surface when the light is incident, and this process is related to the phenomenon of hole-electron generation. The ability to generate this process is achieved by matching the light energy with the bandgap (1.6 eV) of the synthesized photoelectrode, and by exceeding the hot electrons on this value, they are collected over the surface to produce a J_ph_ value. This value is a reflection of the hydrogen moles or rates, or it may indicate the efficiency of the hydrogen gas production.

**Figure 4:**
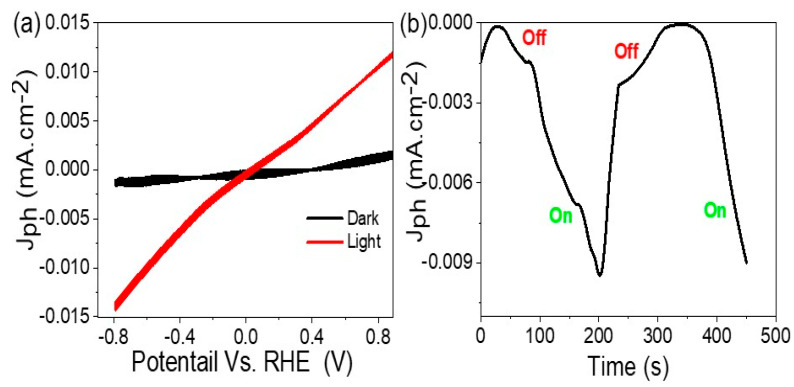
(**a**) The response of the Cr_2_S_3_-Cr_2_O_3_/P2ABT nanocomposite photoelectrode to incident photon under various light conditions from −0.8 to 0.84V and (**b**) under on/off light conditions at −0.8 V.

Figure 4 shows the response of the Cr_2_S_3_-Cr_2_O_3_/P2ABT nanocomposite photoelectrode to incident photons, which is evaluated through J_ph_ values that represent the generated hot electrons. At −0.8 V in Figure 4a, the J_ph_ and J_o_ values are −0.014 and −0.001 mA.cm^−2^, correspondingly, and result in the high sensitivity of the photoelectrode to light compared to dark conditions. The non-linear behavior of the light curve is attributed to the presence of a Schottky barrier [22], which slows down the electron transition between different materials but also confirms that the photoelectrode is the source of the generated electrons.

Figure 4b demonstrates the same behavior of the photoelectrode as in Figure 4a under on/off chopped light, where the large change in J_ph_ values corresponding to the subsequent change in light from on to off indicates the fast and high sensitivity of the photoelectrode to photon behavior. The low Jo values, which are typical for normal semiconductors, indicate that the oxide, sulfide, and polymer materials in the photoelectrode function as effective charge carriers [23,24].

**Figure 5:**
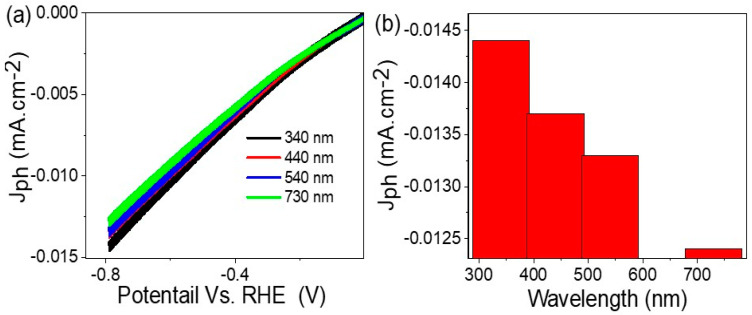
(**a**) The response of the Cr_2_S_3_-Cr_2_O_3_/P2ABT nanocomposite photoelectrode to incident photon under various light frequencies and (**b**) the produced J_ph_ value at −0.8 V.

The hot electron generation process in the Cr_2_S_3_-Cr_2_O_3_/P2ABT nanocomposite photoelectrode is greatly influenced by the photon frequency, illustrated in Figure 5. The energy required for electron transition and crossing the bandgap is a significant factor in this process [25,26,27,28]. The generated hot electron values vary greatly with different photon frequencies (wavelengths). As shown in Figure 5a, as the light frequency increases from 730 to 340 nm, the hot electron values are from −0.014 to −0.012 mA.cm^−2^, respectively. These values are represented in the form of a column shape at −0.8 V (Figure 5b). The wide optical response of the Cr_2_S_3_-Cr_2_O_3_/P2ABT nanocomposite is due to the multiple components that make up the photoelectrode, which work together to enhance the photon sensitivity and electron transition. This wide response is a promising feature for the generation of H_2_ gas since it allows for the efficient use of a broad range of light sources [29,30,31], including natural sunlight. The ability to generate hot electrons and collect them on the surface of the photoelectrode under different frequencies of light also suggests the potential for tuning the performance of the photoelectrode by selecting specific wavelengths of light. This could lead to further optimization of the H_2_ gas generation process and improve its efficiency [32].

**Figure 6:**
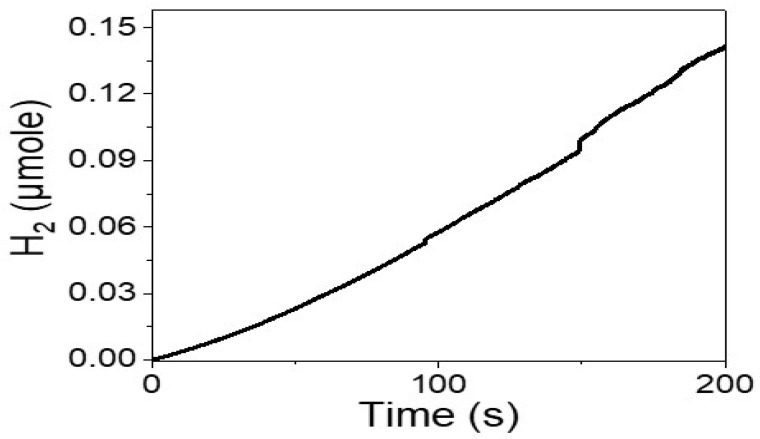
The produced H_2_ moles from sewage water electrolytes using the Cr_2_S_3_-Cr_2_O_3_/P2ABT nanocomposite photoelectrode.

Figure 6 shows the amount of hydrogen gas produced by the Cr_2_S_3_-Cr_2_O_3_/P2ABT/P2ABT nanocomposite photoelectrode under different irradiation times. The efficiency of the photoelectrode is demonstrated by the production of 40.5 mole/10.cm.h of hydrogen gas. This value is significant and shows the potential of using sewage water for hydrogen production. The number of moles of hydrogen gas is calculated using Faraday’s law (Equation (2)) [33], which takes into account the Faraday constant (F) and the molecular weight (M) of hydrogen atoms with an oxidation number of 1.

(2) $$H_{2}\left(\mathrm{moles}\right)=\int_{0}^{t}\frac{J_{ph}dt}{F}.M/z$$

## 4. Conclusions

In summary, this study demonstrates the potential of the Cr_2_S_3_-Cr_2_O_3_/P2ABT nanocomposite photoelectrode as an efficient catalyst for green hydrogen generation. The nanocomposite is synthesized via a one-pot reaction, and its composition is confirmed through XRD and XPS analysis. The nanocomposite exhibits favorable optical properties, with a wide spectral range coverage and a bandgap of 1.6 eV. Electrochemical testing shows an impressive hydrogen production efficiency of 40.5 mole/10.cm.h. The current density values (J_ph_) obtained under different light frequencies using optical filters indicate the nanocomposite’s ability to generate current, highlighting its potential for renewable energy production. The J_ph_ values increase in the wide optical region from 730 to 340 nm, with corresponding hot electron values ranging from −0.014 to −0.012 mA.cm^−2^. Overall, this study presents a promising approach for the utilization of the Cr_2_S_3_-Cr_2_O_3_/P2ABT nanocomposite photoelectrode in green hydrogen generation.

## Figures and Tables

**Table 1:** The positions of the function groups of P2ABT and Cr_2_S_3_-Cr_2_O_3_/P2ABT nanocomposite detected through FTIR analysis.

Band Position (cm^−1^)		Function Group
Cr_2_S_3_-Cr_2_O_3_/P2ABT	P2AMB	
3760	3743	N-H
3354	3373	S-H [12] and O-H
1600	1621	quinoid C=C
1473	1514	benzene C=C
1294	1304	C-N
1131	1126	C-H
756	757	Para disubstituted ring
609	593	C-H out of plane

**Table 2:** Concentration (mg/L) for anions, compounds, and heavy metals in the sewage water used for the electrochemical H_2_ generation.

Material	Conc, (mg/L)
F^-^	1.0
CN^−1^	0.1
Phenols	0.015
Pb^2+^	0.5
Al^3+^	3.0
Cd^3+^	0.05
NH_3_	5.0
Cr^3+^	1.0
Hg^2+^	0.005
Mn^2+^	1.0
As^3+^	0.05
Ba^3+^	2.0
Cu^2+^	1.5
Co^2+^	2.0
Ni^3+^	0.1
Fe^3+^	1.5
Ag^+^	0.1
Zn^2+^	5.0
Other cations	0.1
Pesticides	0.2
Coli groups	4000/100 cm^3^
Industrial washing	0.5

## Data Availability

All data generated or analyzed during this study are included in this article.
